# Comparison of Choroidal Morphological Changes Between Aflibercept 8 mg and Faricimab-svoa in Treatment-Naïve Polypoidal Choroidal Vasculopathy and Pachychoroid Neovasculopathy

**DOI:** 10.3390/jcm15114355

**Published:** 2026-06-04

**Authors:** Seongyong Jeong, Seung Hyeon Seong, Min Sagong

**Affiliations:** 1 Department of Ophthalmology, Yeungnam University College of Medicine, Daegu 42415, Republic of Korea; seongyong.jeong@yu.ac.kr (S.J.); win7656@naver.com (S.H.S.); 2 Yeungnam Eye Center, Yeungnam University Hospital, Daegu 42415, Republic of Korea

**Keywords:** pachychoroid neovasculopathy, polypoidal choroidal vasculopathy, aflibercept 8 mg, faricimab-svoa, choroidal vascularity index, Ang-2 inhibition

## Abstract

**Background/Objectives**: This study compared choroidal morphological changes, including choroidal vascularity index (CVI) and layer-specific choroidal thickness, between aflibercept 8 mg and faricimab-svoa in treatment-naïve polypoidal choroidal vasculopathy (PCV) and pachychoroid neovasculopathy (PNV). **Methods**: This retrospective study included 66 eyes treated with aflibercept 8 mg (*n* = 32) or faricimab-svoa (*n* = 34). Following three monthly loading injections, a pro re nata regimen was employed for 6 months. Best-corrected visual acuity (BCVA), central macular thickness (CMT), subfoveal choroidal thickness (SFCT), pigment epithelial detachment (PED) height, and CVI were assessed. **Results**: Both groups demonstrated significant improvements in BCVA, CMT, SFCT, and PED height at 6 months (all *p* < 0.05), with no between-group differences. Dry macula rates were 75.0% and 79.4%, respectively. Faricimab-svoa was associated with a significantly greater CVI increase (0.037 vs. 0.018, *p* = 0.003), driven by a numerically greater reduction in choriocapillaris/Sattler’s layer thickness (−18.9 ± 16.4 μm vs. −13.8 ± 15.8 μm, *p* = 0.153). **Conclusions**: Both agents achieved comparable functional and anatomical outcomes in treatment-naïve PCV and PNV. Faricimab-svoa was associated with a greater CVI increase, reflecting differential choroidal remodeling. CVI may serve as a biomarker for differentiating the choroidal effects of second-generation anti-VEGF therapies in pachychoroid spectrum disease.

## 1. Introduction

The pachychoroid spectrum encompasses a group of conditions sharing a common structural hallmark: pathological dilation of the outer choroidal (Haller’s) layer vessels with attenuation of the inner Sattler’s and choriocapillaris layers [1,2,3]. Among these, pachychoroid neovasculopathy (PNV) is defined as type 1 macular neovascularization (MNV) arising in the setting of pachychoroid features, whereas polypoidal choroidal vasculopathy (PCV) represents aneurysmal type 1 MNV characterized by a branching neovascular network with polypoidal terminal dilation [1,4,5,6,7,8].

The pathophysiology of pachychoroid-associated neovascularization is driven by chronic choroidal venous congestion, frequently involving vortex vein stasis and intervortex venous anastomoses, which leads to choriocapillaris ischemia and choroidal hyperpermeability [9,10]. In this hemodynamically stressed microenvironment, decreased oxygen diffusion from the choriocapillaris to the outer retina may promote vascular endothelial growth factor (VEGF) expression from the retinal pigment epithelium (RPE) [2]. In parallel, aqueous humor angiopoietin-2 (Ang-2) levels have been reported to be significantly elevated in pachychoroid-associated MNV compared to controls [11]. Together, these two mediators may destabilize the inner choroidal vasculature, promote vascular leakage into the surrounding stroma, and create the pro-angiogenic milieu that underpins type 1 MNV.

Two second-generation intravitreal agents have been developed to address the limitations of first-generation anti-VEGF monotherapy. Faricimab-svoa, the first bispecific antibody for intraocular use, independently neutralizes both VEGF-A and Ang-2, targeting the dual pathways implicated in pachychoroid-driven vascular instability [12,13,14]. Aflibercept 8 mg delivers a higher molar dose of VEGF blockade, which extended treatment durability compared with aflibercept 2 mg in the PULSAR trial [15].

Quantitative choroidal assessment is essential for understanding the therapeutic response in pachychoroid diseases. Although subfoveal choroidal thickness (SFCT) has been widely used, it does not distinguish vascular from stromal compartmental changes. The choroidal vascularity index (CVI), defined as the ratio of luminal to total choroidal area, provides this compartmental resolution and has emerged as a more mechanistically informative biomarker [16]. Whether the distinct molecular mechanisms of these two agents may translate into different patterns of choroidal remodeling—detectable by CVI—have not been directly examined in PNV and PCV [17,18].

This study aimed to compare functional and anatomical outcomes, including choroidal morphological changes assessed by CVI and layer-specific choroidal thickness, between aflibercept 8 mg and faricimab-svoa in treatment-naïve patients with PNV and PCV.

## 2. Materials and Methods

### 2.1. Study Design and Participants

All procedures performed in this study were in accordance with the ethical standards of the Helsinki Declaration. The study was approved by the Institutional Review Board (IRB) of Yeungnam University Hospital (2026-02-009). Data collection and analyses were initiated after IRB approval was obtained. Written informed consent was waived by the IRB due to the retrospective nature of the study. The retrospective comparative study encompassed consecutive patients treated between March 2024 and July 2025. Eligible patients were treatment-naïve individuals aged ≥50 years with a confirmed diagnosis of active PCV or PNV with subfoveal or fovea-involving neovascular lesions and a minimum follow-up of 6 months. Active disease was defined as any evidence of exudative activity on OCT, including subretinal fluid (SRF) and/or intraretinal fluid (IRF). PCV was diagnosed based on the presence of polypoidal lesions on indocyanine green angiography (ICGA) in accordance with the Asia-Pacific Ocular Imaging Society PCV Workgroup criteria [4]. PNV was diagnosed by the presence of type 1 MNV on optical coherence tomography (OCT) in the context of pachychoroid features without discrete polypoidal lesions. Exclusion criteria included: prior intravitreal anti-VEGF injection, photodynamic therapy, or laser photocoagulation; concurrent retinal disorders such as pathologic myopia, retinal vein occlusion, or diabetic retinopathy; and a history of intraocular surgery other than uncomplicated cataract surgery.

### 2.2. Treatment Protocol

The choice between aflibercept 8 mg and faricimab-svoa was determined by the treating retinal specialist (M.S.). Because the two agents were considered to have broadly comparable efficacy, allocation was not based on individual patient demographics, baseline visual acuity, or lesion characteristics. All patients received three initial monthly loading injections (at baseline, month 1, and month 2) of either aflibercept 8 mg/0.07 mL (Eylea HD; Regeneron Pharmaceuticals, Tarrytown, NY, USA/Bayer AG, Leverkusen, Germany) or faricimab-svoa 6 mg/0.05 mL (Vabysmo; Genentech, Inc., South San Francisco, CA, USA/F. Hoffmann-La Roche Ltd., Basel, Switzerland). Following the loading phase, a pro re nata (PRN) regimen was employed from month 3 through month 6. Retreatment was indicated by any of the following criteria: persistent or recurrent SRF or IRF on OCT; new or recurrent hemorrhage; or a decrease in best-corrected visual acuity (BCVA) attributable to fluid.

### 2.3. Ophthalmologic Examinations and Imaging

All patients underwent comprehensive ophthalmologic evaluation at baseline and at monthly intervals thereafter. BCVA was converted to logarithm of the minimum angle of resolution (logMAR) for analysis. Quantitative OCT measurements were performed independently by two graders (S.J. and S.H.S.) who were masked to the treatment group and visit time point, and the mean of the two measurements was used in the analysis. Central macular thickness (CMT) was defined as the mean retinal thickness within the foveal-centered 1 mm diameter circle of the Early Treatment Diabetic Retinopathy Study (ETDRS) grid, derived from the integrated macular thickness map of the Spectralis OCT (Heidelberg Engineering, Heidelberg, Germany). SFCT was measured perpendicularly from Bruch’s membrane to the choroidal–scleral interface. Layer-specific choroidal thickness was measured for the Haller’s layer (outer large-vessel layer) and the combined choriocapillaris/Sattler’s layer (inner medium- and small-vessel layer). Pigment epithelial detachment (PED) height was measured as the maximum vertical distance from the base of Bruch’s membrane to the apex of the elevated RPE. Dry macula was defined as the complete resolution of all IRF and SRF on OCT.

### 2.4. CVI Analysis

The CVI was quantified as the ratio of the luminal choroidal area (LCA) to the total choroidal area (TCA) using subfoveal enhanced depth imaging-OCT (EDI-OCT) B-scans acquired with an 8.8 mm scan width. For each eye, a single horizontal B-scan passing through the foveal center was selected for analysis. Image quality was confirmed by visual inspection to ensure clear visualization of the choroidal–scleral interface, and scans with insufficient signal strength or significant motion artifacts were excluded. To ensure measurement consistency across timepoints, the Spectralis OCT follow-up function (AutoRescan with TruTrack eye tracking) was used to acquire baseline and follow-up scans at the identical anatomical location. Selected EDI-OCT B-scans were processed using ImageJ version 1.54p (National Institutes of Health, Bethesda, MD, USA) with a modified Niblack local binarization algorithm to differentiate luminal (dark pixel, vessels) from stromal (light pixel, interstitium) components. The TCA was delineated from the basal margin of the RPE to the inner scleral surface (see Figure S1 in the Supplementary Material). CVI measurements were independently performed by two graders (S.J. and S.H.S.) using ImageJ on exported EDI-OCT images, with masking to both treatment group and imaging timepoint, following a predefined, standardized protocol, and the mean of the two measurements was used in the analysis.

### 2.5. Polyp Closure Assessment

In the PCV subgroup, polypoidal lesion (PL) response to treatment was assessed using OCT alone, without confirmatory ICGA, based on the validated OCT classification system described by Chaikitmongkol et al. [19]. PED morphology at each PL site was categorized into five prespecified features: feature A, no PED; feature B, PED with internal homogeneous reflectivity with predominant blended RPE-underlying structure (BUN) sign; feature C, PED with internal homogeneous reflectivity with minimal BUN sign; feature D, heterogeneous PED; and feature E, PED with hyporeflectivity. Complete polyp closure was defined as OCT features A or B (complete resolution of the hyporeflective lumen within the PED, with flattening or homogeneous hyperreflective filling). Partial closure was defined as features C, and no response as persistent feature D (heterogeneous PED with internal hyporeflectivity). Assessment was performed independently by two retinal specialists (S.J., M.S.) at the post-loading phase visit (month 3), and discrepancies were resolved by consensus.

### 2.6. Statistical Analysis

Statistical analyses were performed using SPSS version 24.0 (IBM Corp., Armonk, NY, USA). Continuous variables are presented as mean ± standard deviation. The normality of continuous variables was assessed using the Shapiro–Wilk test. For between-group comparisons of continuous variables, Welch’s *t*-test was used when the normality assumption was satisfied, and the Mann–Whitney U test was used when the normality assumption was violated. For within-group comparisons between baseline and 6-month values, the paired *t*-test was used for normally distributed variables, and the Wilcoxon signed-rank test was used for non-normally distributed variables. Categorical variables were compared using the chi-square test or Fisher’s exact test, as appropriate. A *p*-value < 0.05 was considered statistically significant. Inter-grader reliability for OCT-based measurements (SFCT, Haller’s layer thickness, choriocapillaris/Sattler’s layer thickness, PED height) and CVI was assessed using two-way mixed-effects absolute-agreement intraclass correlation coefficients (ICCs).

## 3. Results

### 3.1. Baseline Characteristics

A total of 66 eyes from 66 patients with PCV (*n* = 34) or PNV (*n* = 32) were enrolled. Patients were grouped into the aflibercept 8 mg group (*n* = 32) and the faricimab-svoa group (*n* = 34). The aflibercept 8 mg group comprised 17 eyes with PCV and 15 eyes with PNV, while the faricimab-svoa group comprised 17 eyes with PCV and 17 eyes with PNV (*p* = 0.811). Mean age was 69.3 ± 9.2 years in the aflibercept 8 mg group and 68.3 ± 7.3 years in the faricimab-svoa group (*p* = 0.620). Baseline BCVA, CMT, SFCT, Haller’s layer thickness, choriocapillaris/Sattler’s layer thickness, PED height, and CVI did not differ significantly between groups. The proportions of eyes with SRF or IRF were also comparable (all *p* > 0.05; Table 1). Inter-grader reliability for all OCT-based measurements was excellent, with ICCs of 0.94 for SFCT, 0.93 for Haller’s layer thickness, 0.90 for choriocapillaris/Sattler’s layer thickness, 0.95 for PED height, and 0.94 for CVI.

### 3.2. Functional and Anatomical Outcomes at 6 Months

During the 6-month observation period, the aflibercept 8 mg group received a mean of 3.53 ± 0.72 injections and the faricimab-svoa group received 3.32 ± 0.68 injections (*p* = 0.234). A high rate of dry macula was achieved after the loading phase: 26 of 32 eyes (81.3%) in the aflibercept 8 mg group and 29 of 34 eyes (85.3%) in the faricimab-svoa group (*p* = 0.748). At 6 months, dry macula was maintained in 24 of 32 (75.0%) and 27 of 34 (79.4%) eyes, respectively (*p* = 0.772).

**Figure 1:**
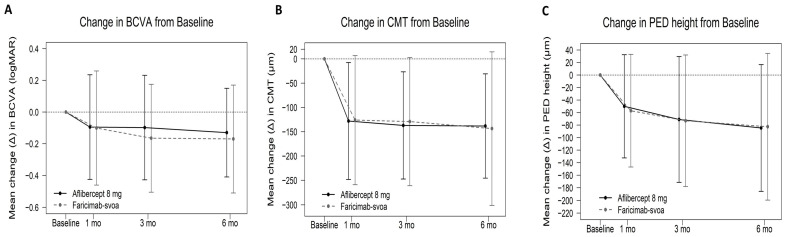
Mean changes from baseline in BCVA, CMT, and PED height over 6 months in the aflibercept 8 mg (*n* = 32) and faricimab-svoa (*n* = 34) groups. (**A**) Mean change in best-corrected visual acuity (BCVA, logMAR); (**B**) mean change in central macular thickness (CMT, μm); (**C**) mean change in pigment epithelial detachment (PED) height (μm). Data points represent the mean value at each timepoint, and error bars represent the standard deviation (SD).

BCVA improved significantly in both groups (from 0.53 ± 0.48 to 0.40 ± 0.36 logMAR, *p* = 0.017; and from 0.55 ± 0.53 to 0.38 ± 0.34 logMAR, *p* = 0.006, respectively). CMT decreased significantly in both groups (*p* < 0.001 for both). Mean PED height was reduced in both groups (from 253.3 ± 148.9 to 168.7 ± 125.7 μm and from 205.9 ± 175.8 to 123.4 ± 95.0 μm, respectively; both *p* < 0.001; Figure 1). SFCT decreased to 277.3 ± 71.8 μm in the aflibercept 8 mg group (*p* < 0.001) and 276.7 ± 64.8 μm in the faricimab-svoa group (*p* < 0.001). At the layer-specific level, Haller’s layer thickness decreased significantly in both groups (to 206.7 ± 50.6 μm and 205.1 ± 52.6 μm, respectively; both *p* < 0.001). choriocapillaris/Sattler’s layer thickness also decreased significantly in both groups (to 70.2 ± 27.6 μm and 68.6 ± 21.9 μm; *p* = 0.003 and *p* < 0.001, respectively). CVI increased significantly in both groups, from 0.678 ± 0.016 to 0.696 ± 0.020 in the aflibercept 8 mg group (*p* < 0.001), and from 0.674 ± 0.023 to 0.712 ± 0.033 in the faricimab-svoa group (*p* < 0.001; Table 2).

RPE tears occurred in 1 eye (3.1%) in the aflibercept 8 mg group and 2 eyes (5.9%) in the faricimab-svoa group; all three affected eyes had a baseline diagnosis of PCV. In all cases, the RPE tear was detected at the 1-month visit following the first loading injection. Baseline PED heights of the affected eyes were 501 μm, 630 μm, and 577 μm. Baseline BCVA of the affected eyes was 1.00, 1.40, and 1.00 logMAR, respectively. At the 6-month visit, BCVA was 0.82, 0.40, and 1.10 logMAR, and PED height had decreased to 170 μm, 332 μm, and 289 μm, respectively. No cases of intraocular inflammation or retinal vasculitis, and no other ocular or systemic adverse events, were observed during the 6-month follow-up period in either group.

### 3.3. Between-Group Comparison of Changes

**Figure 2:**
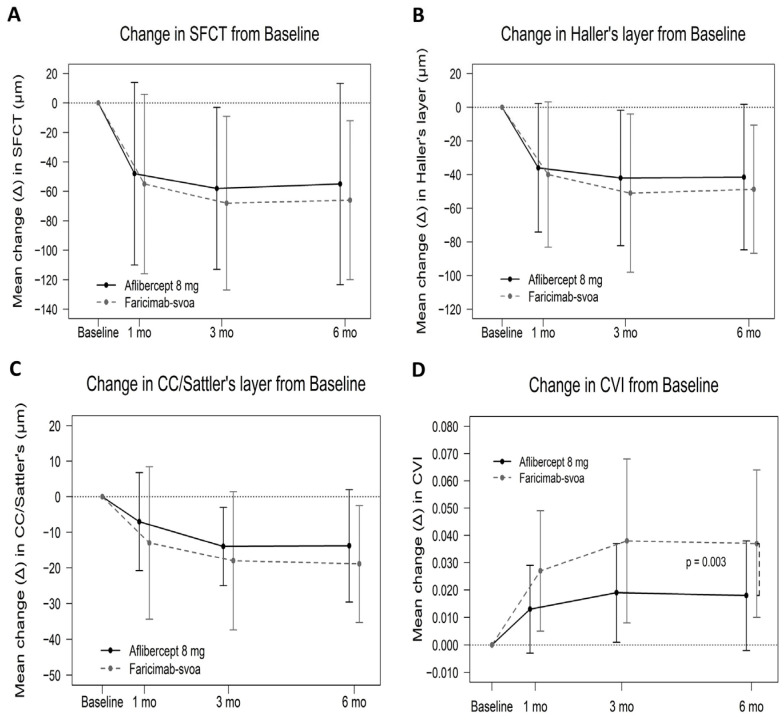
Mean changes in choroidal thickness (SFCT, Haller’s layer, and choriocapillaris/Sattler’s layer), and CVI from baseline over 6 months in the aflibercept 8 mg (*n* = 32) and faricimab-svoa (*n* = 34) groups. (**A**) Mean change in subfoveal choroidal thickness (SFCT, μm); (**B**) mean change in Haller’s layer thickness (μm); (**C**) mean change in choriocapillaris (CC)/Sattler’s layer thickness (μm); (**D**) mean change in choroidal vascularity index (CVI, %). Data points represent the mean value at each timepoint, and error bars represent the standard deviation (SD). The between-group difference in ΔCVI at 6 months was statistically significant (*p* = 0.003).

**Figure 3:**
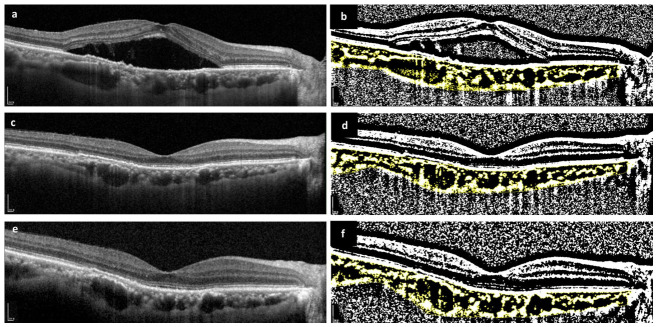
Representative case of faricimab-svoa treatment in a 68-year-old female patient with pachychoroid neovasculopathy. (**a**,**b**) Baseline: Enhanced depth imaging-optical coherence tomography (EDI-OCT) and its corresponding binarized image reveal prominent subretinal fluid (SRF) and a baseline choroidal vascularity index (CVI) of 0.671. (**c**,**d**) 3 months: After three monthly loading injections of faricimab-svoa, the SRF has completely resolved, achieving a dry macula. The CVI has significantly increased to 0.707, reflecting effective choroidal stromal remodeling. Although the luminal area decreased, the stromal compartment showed a relatively greater reduction, resulting in an increased CVI. (**e**,**f**) 6 months: The anatomical improvement is well-maintained through the 6-month follow-up without additional injections. Although a slight increase in overall choroidal thickness was observed, the CVI (0.701) remained increased compared with baseline.

**Figure 4:**
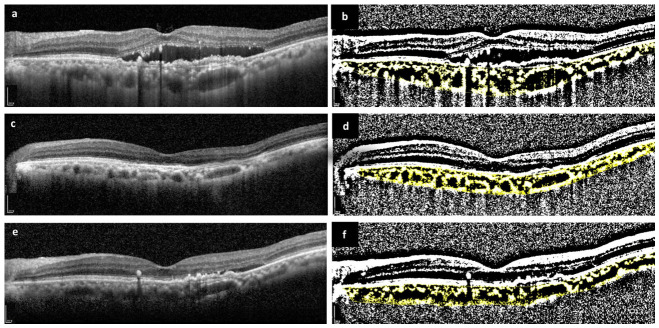
Representative case of aflibercept 8 mg treatment in a 78-year-old male patient with pachychoroid neovasculopathy. (**a**,**b**) Baseline: Enhanced depth imaging-optical coherence tomography (EDI-OCT) and its corresponding binarized image show significant subretinal fluid (SRF). The baseline choroidal vascularity index (CVI) is 0.652. (**c**,**d**) 3 months: Following three monthly loading injections of aflibercept 8 mg, the SRF has completely resolved, achieving a dry macula. However, although the luminal area decreased, the reduction in the stromal compartment was relatively insufficient, resulting in a slight decrease in CVI (0.648). (**e**,**f**) 6 months: At the 6-month follow-up, a recurrence of SRF is observed. Enlargement of a large choroidal vessel was noted, along with a concurrent increase in the stromal compartment, and the CVI remained unchanged (0.650).

Between-group comparison revealed no statistically significant differences in ΔBCVA, ΔCMT, ΔSFCT, ΔHaller’s layer, or ΔPED height. The reduction in choriocapillaris/Sattler’s layer thickness was numerically greater in the faricimab-svoa group (−18.9 ± 16.4 vs. −13.8 ± 15.8 μm), although this did not reach statistical significance (*p* = 0.153). The CVI increase was significantly greater in the faricimab-svoa group (ΔCVI: 0.037 ± 0.027 vs. 0.018 ± 0.020, *p* = 0.003). The ΔTCA and ΔLCA did not differ significantly between groups (*p* = 0.772 and *p* = 0.818, respectively; Table 3; Figure 2). Representative cases illustrating the distinct choroidal remodeling patterns are shown in Figure 3 and Figure 4.

### 3.4. Subgroup Analysis: Polyp Closure in PCV

Among the 34 eyes with PCV, complete polyp closure after the loading phase was achieved in 6 of 17 eyes (35.3%) in the aflibercept 8 mg group and 8 of 17 eyes (47.1%) in the faricimab-svoa group (*p* = 0.492). Combined complete and partial polyp closure rates were 70.6% and 76.5%, respectively (*p* = 0.702). No response was observed in 5 (29.4%) and 4 (23.5%) eyes in each group, respectively (Table 4). The inter-grader reliability using a Cohen’s kappa value was 0.911.

## 4. Discussion

This retrospective comparative study evaluated the 6-month choroidal and functional effects of two second-generation intravitreal agents—aflibercept 8 mg and faricimab-svoa—in treatment-naïve patients with PCV and PNV. Both agents demonstrated significant improvements in BCVA, CMT, PED height, and SFCT, with no significant between-group differences in these parameters. The principal finding of the study was a significantly greater increase in CVI in the faricimab-svoa group (ΔCVI: 0.037 vs. 0.018; *p* = 0.003), accompanied by a numerically greater reduction in choriocapillaris/Sattler’s layer thickness, suggesting that dual Ang-2/VEGF-A inhibition confers a qualitatively distinct pattern of choroidal remodeling that extends beyond the shared anti-VEGF effect.

The functional and anatomical improvements observed in our cohort are consistent with those reported in large-scale clinical trials and real-world studies of both agents in neovascular AMD [15,20,21,22,23,24]. In the current study, a high rate of macular drying was achieved after the loading phase—81.3% in the aflibercept 8 mg group and 85.3% in the faricimab-svoa group—aligned with dry macula rates of 57–82% reported in previous studies of second-generation agents [25,26,27]. At 6 months, dry macula was maintained in 75.0% and 79.4% of eyes, respectively (*p* = 0.772), demonstrating sustained fluid control under a PRN regimen with both agents.

Among the 34 PCV eyes, the complete polyp closure rate after the loading phase was 35.3% in the aflibercept 8 mg group and 47.1% in the faricimab-svoa group (*p* = 0.492), with combined complete and partial closure rates of 70.6% and 76.5%, respectively (*p* = 0.702). These rates are broadly consistent with the published literature for faricimab-svoa in PCV: a recent systematic review and meta-analysis by Arnold-Vangsted et al. reported a pooled complete polyp closure rate of 48.7% (95% CI: 32.5–65.0%) after faricimab-svoa loading in treatment-naïve eyes [28]. Our result for the faricimab-svoa group (47.1% complete closure) aligns closely with this pooled estimate. For the aflibercept 8 mg group, direct comparative data in PCV remain limited; the polyp closure rate of 35.3% observed here is within the range of 32.4–59% reported for aflibercept 2 mg in previous real-world studies [29,30,31], and the numerically higher complete closure rate with faricimab-svoa (47.1% vs. 35.3%) is consistent with the 1-year real-world comparative data of Cho et al., who found no statistically significant difference in polyp response between faricimab-svoa and aflibercept, although a numerical trend toward higher polyp closure with faricimab-svoa was noted [29]. These findings collectively suggest that both second-generation agents provide comparable overall polyp control, with faricimab-svoa potentially offering a modest advantage in polyp closure that warrants confirmation in larger, ICGA-confirmed prospective cohorts. As polyp activity in our study was assessed by OCT morphology alone, these closure rates should be interpreted as estimates of OCT-defined polyp regression. The phase IIIb/IV SALWEEN trial recently reported a 51% reading center–confirmed complete polyp regression rate after four monthly loading doses of faricimab-svoa monotherapy in treatment-naïve PCV [32].

Both agents significantly reduced SFCT, with mean decreases of −55.2 μm in the aflibercept 8 mg and −65.8 μm in the faricimab-svoa groups, without a significant between-group difference (*p* = 0.389). The degree of choroidal thinning likely reflects attenuation of choroidal vascular engorgement and hyperpermeability [2,33,34]. More importantly, CVI increased significantly in both groups (ΔCVI: 0.018 in the aflibercept 8 mg group and 0.037 in the faricimab-svoa group; both *p* < 0.001), and this increase was significantly greater in the faricimab-svoa group (*p* = 0.003). Because ΔTCA and ΔLCA were statistically comparable between groups (*p* = 0.772 and *p* = 0.818), the greater CVI rise in the faricimab-svoa group was driven by a preferential reduction in the choroidal stromal (extravascular) compartment rather than differential vascular effects. This observation is consistent with the findings of Jeong et al., who similarly demonstrated a significantly greater CVI increase with brolucizumab compared with aflibercept 2 mg in treatment-naïve PNV, attributing the difference to preferential reduction in stromal edema by the higher-penetration agent [17]. Taken together, these data suggest that CVI—by segregating luminal from stromal compartmental changes—may be a more sensitive indicator of drug-specific choroidal remodeling than SFCT alone, particularly in pachychoroid diseases where stromal hyperpermeability is a central pathological feature.

The differential CVI response most plausibly reflects the distinct molecular mechanisms of the two agents. Aflibercept 8 mg, acting as a high-affinity VEGF trap, effectively suppresses VEGF-driven vascular leakage and outer choroidal engorgement—consistent with the comparable Haller’s layer reductions seen in both groups (−41.5 vs. −49.7 μm, *p* = 0.452). However, VEGF suppression alone does not address Ang-2-mediated vascular destabilization. Ang-2 levels in aqueous humor have been reported to be significantly elevated in pachychoroid-associated MNV compared to controls [11]. Elevated Ang-2 competitively antagonizes the Ang-1/TEK (Tie2) axis: Ang-1, secreted by PDGFRB-positive pericytes and stromal fibroblasts, normally activates TEK on adjacent choroidal endothelial cells to maintain PI3K/Akt-mediated barrier integrity [35,36]. Ang-2 displacement of Ang-1 from TEK causes pericyte dropout, endothelial junction disassembly, and plasma extravasation into the choroidal stroma—expanding the stromal compartment and reducing CVI [37]. Faricimab-svoa, by additionally neutralizing Ang-2, may restore Ang-1/TEK signaling and resolve this stromal edema, accounting for its greater CVI gain without a corresponding difference in ΔLCA (*p* = 0.818).

The layer-specific data support this interpretation. Although not statistically significant, a numerically greater choriocapillaris/Sattler’s layer reduction was observed with faricimab-svoa than with aflibercept 8 mg (−18.9 vs. −13.8 μm, *p* = 0.153). Because these layers have a lower luminal-to-stromal ratio than the Haller’s layer, resolution of perivascular stromal edema at this level produces a disproportionate CVI rise relative to the luminal change—explaining why faricimab-svoa’s CVI advantage exceeds what its modest additional Sattler’s layer thinning alone would predict. By contrast, aflibercept 8 mg achieves comparable outer choroidal decompression through VEGF blockade but less inner-layer stromal resolution, yielding a smaller yet still significant CVI gain. Together, these findings suggest that the two agents remodel the choroid through partially overlapping but mechanistically distinct pathways, and that CVI—by separating luminal from stromal changes—captures this distinction more sensitively than SFCT alone.

The comparable reduction in PED height between the two groups (−84.5 vs. −82.5 μm; *p* = 0.940) is consistent with the findings of Mukai et al., who reported similar PED regression between faricimab-svoa and aflibercept 2 mg in a real-world cohort [38]. The modestly superior PED reduction with faricimab-svoa observed in the TENAYA/LUCERNE post hoc analysis (approximately 10 μm at 6 months) [39] was not reproduced in the present study comparing second-generation agents, suggesting that any incremental Ang-2-mediated benefit in PED resolution may be attenuated when the comparator is the higher-dose aflibercept 8 mg formulation.

This study has several limitations that should be considered when interpreting the findings. First, the retrospective, single-center, non-randomized design introduces potential selection bias and limits causal inference; the absence of randomization means that unmeasured confounders—including baseline disease severity and physician preference—cannot be excluded. Second, the relatively small sample size (32 and 34 eyes per group) may reduce statistical power, particularly for subgroup analyses such as polyp closure in PCV and layer-specific choroidal measurements. Third, the 6-month follow-up period is insufficient to assess the long-term durability of choroidal remodeling or the potential clinical impact of early CVI differences on visual outcomes, disease recurrence, or treatment interval extension. Fourth, the CVI analysis was performed on a single subfoveal EDI-OCT B-scan using a two-dimensional manual binarization method, which may not fully represent three-dimensional volumetric choroidal changes; swept-source OCT-based volumetric CVI assessment would provide more comprehensive data. Fifth, polyp closure was assessed by OCT morphology alone without confirmatory ICGA at the post-loading visit, which may have led to under- or overestimation of true polyp regression rates, given that OCT-based assessment has inherent limitations in detecting residual polypoidal activity compared with ICGA. Sixth, inter-observer variability in CVI binarization and polyp closure grading, although mitigated by consensus review, remains a potential source of measurement error. Seventh, the study did not include OCT angiography (OCTA) analysis, which could have provided complementary information on choriocapillaris flow changes and their relationship with CVI alterations. Eighth, although PCV and PNV share pachychoroid-related choroidal abnormalities, they are distinct clinical phenotypes; therefore, the pooled analysis should be interpreted with caution. Finally, this study was exploratory in nature and involved testing of multiple variables without formal correction for multiple comparisons; consequently, the possibility of type I error should be acknowledged. These limitations highlight the need for large-scale, prospective, ICGA-confirmed, randomized studies with volumetric choroidal imaging and extended follow-up to validate and expand upon the current findings.

## 5. Conclusions

In summary, both aflibercept 8 mg and faricimab-svoa demonstrated favorable and comparable functional and anatomical outcomes in treatment-naïve patients with PCV and PNV over a 6-month follow-up period. Both agents achieved high rates of macular drying and comparable OCT-defined polyp closure in PCV, with no significant between-group differences in BCVA, CMT, SFCT, or PED height. The principal distinguishing finding was a significantly greater increase in CVI in the faricimab-svoa group, driven by preferential reduction in the choroidal stromal compartment and accompanied by a trend toward greater inner (choriocapillaris/Sattler’s) layer thinning. These observations suggest that dual Ang-2/VEGF-A inhibition confers an additional anti-permeability effect on the pachychoroid stroma beyond VEGF suppression alone, consistent with the mechanistic role of Ang-2-driven endothelial hyperpermeability in this disease spectrum. CVI, by enabling compartmental separation of vascular and stromal changes, may serve as a sensitive biomarker for differentiating the choroidal remodeling profiles of second-generation anti-VEGF agents—a distinction not captured by SFCT alone. The clinical implication is that agents targeting both VEGF and Ang-2 may offer qualitatively different, and potentially complementary, choroidal effects compared with high-dose VEGF monotherapy; whether this translates into superior long-term outcomes in terms of disease durability, treatment interval, or prevention of choroidal atrophy remains to be determined. Large-scale, prospective, randomized studies with volumetric choroidal imaging, ICGA-confirmed polyp assessment, and extended follow-up are warranted to validate these early mechanistic insights and to clarify the role of CVI-guided treatment monitoring in pachychoroid spectrum disease.

## Figures and Tables

**Table 1:** Baseline characteristics of eyes treated with aflibercept 8 mg or faricimab-svoa.

	Aflibercept 8 mg (*n* = 32)	Faricimab-svoa (*n* = 34)	*p*-Value
Age (years)	69.3 ± 9.2	68.3 ± 7.3	0.620 ^a^
Male sex, *n* (%)	22 (68.8%)	21 (61.8%)	0.552
BCVA (logMAR)	0.53 ± 0.48	0.55 ± 0.53	0.882 ^b^
Hypertension, *n* (%)	12 (37.5%)	16 (47.1%)	0.432
Diabetes mellitus, *n* (%)	5 (15.6%)	9 (26.5%)	0.281
PCV/PNV	17/15	17/17	0.811
IRF presence, *n* (%)	10 (31.3%)	6 (17.6%)	0.255
SRF presence, *n* (%)	31 (96.9%)	32 (94.1%)	0.557
PED height (μm)	253.3 ± 148.9	205.9 ± 175.8	0.241 ^a^
CMT (μm)	448.0 ± 138.5	419.6 ± 136.7	0.408 ^b^
SFCT (μm)	332.5 ± 84.9	342.4 ± 77.7	0.625 ^a^
Haller’s layer (μm)	248.0 ± 61.1	254.9 ± 55.3	0.682 ^a^
Choriocapillaris/Sattler’s layer (μm)	84.0 ± 29.4	87.5 ± 30.4	0.631 ^b^
CVI	0.678 ± 0.016	0.674 ± 0.023	0.494 ^a^
TCA (mm^2^)	1.904 ± 0.539	1.914 ± 0.510	0.871 ^b^
LCA (mm^2^)	1.276 ± 0.335	1.289 ± 0.202	0.854 ^b^
Greatest linear dimension (μm)	2949.5 ± 1352.9	3037.8 ± 958.9	0.762 ^b^
Polyp number	2.00 ± 1.87	2.06 ± 1.85	0.927
Largest polyp diameter (μm)	245.1 ± 85.9	231.7 ± 64.1	0.610 ^b^

BCVA = best-corrected visual acuity; CMT = central macular thickness; CVI = choroidal vascularity index; IRF = intraretinal fluid; LCA = luminal choroidal area; LogMAR = logarithm of the minimum angle of resolution; PCV = polypoidal choroidal vasculopathy; PED = pigment epithelial detachment; PNV = pachychoroid neovasculopathy; SFCT = subfoveal choroidal thickness; SRF = subretinal fluid; TCA = total choroidal area. ^a^ *p*-value using Welch’s *t*-test. ^b^ *p*-value using the Mann–Whitney U test. Continuous variables are presented as mean ± standard deviation and were compared using the independent *t*-test. Categorical variables are presented as *n* (%) and were compared using the chi-square test or Fisher’s exact test. A *p*-value < 0.05 was considered statistically significant.

**Table 2:** Functional and anatomical outcomes at 6 months in the aflibercept 8 mg and faricimab-svoa groups.

	Aflibercept 8 mg (*n* = 32)			Faricimab-svoa (*n* = 34)		
	Baseline	6 mo	*p*-Value	Baseline	6 mo	*p*-Value
BCVA (logMAR)	0.53 ± 0.48	0.40 ± 0.36	0.017 ^b^	0.55 ± 0.53	0.38 ± 0.34	0.006 ^b^
CMT (μm)	448.0 ± 138.5	309.8 ± 98.3	<0.001 ^a^	419.6 ± 136.7	276.3 ± 97.1	<0.001 ^a^
SFCT (μm)	332.5 ± 84.9	277.3 ± 71.8	<0.001 ^a^	342.7 ± 77.7	276.7 ± 64.8	<0.001 ^a^
Haller layer (μm)	248.0 ± 61.1	206.7 ± 50.6	<0.001 ^b^	254.9 ± 55.3	205.1 ± 52.6	<0.001 ^b^
Choriocapillaris/Sattler layer (μm)	84.0 ± 29.4	70.2 ± 27.6	0.003 ^b^	87.5 ± 30.4	68.6 ± 21.9	<0.001 ^b^
PED height (μm)	253.2 ± 148.9	168.7 ± 125.7	<0.001 ^b^	205.9 ± 175.8	123.4 ± 95.0	<0.001 ^b^
IRF presence, *n* (%)	10 (31.2%)	2 (6.3%)	0.008	6 (17.6%)	1 (2.9%)	0.062
SRF presence, *n* (%)	31 (96.9%)	7 (21.9%)	<0.001	32 (94.1%)	7 (20.6%)	<0.001
CVI	0.678 ± 0.016	0.696 ± 0.020	<0.001 ^a^	0.674 ± 0.023	0.712 ± 0.033	<0.001 ^a^
TCA (mm^2^)	1.904 ± 0.539	1.770 ± 0.410	0.156 ^a^	1.914 ± 0.510	1.759 ± 0.367	0.026 ^a^
LCA (mm^2^)	1.276 ± 0.335	1.222 ± 0.381	0.133 ^a^	1.289 ± 0.202	1.243 ± 0.496	0.182 ^a^

BCVA = best-corrected visual acuity; CMT = central macular thickness; CVI = choroidal vascularity index; IRF = intraretinal fluid; LCA = luminal choroidal area; LogMAR = logarithm of the minimum angle of resolution; PED = pigment epithelial detachment; SFCT = subfoveal choroidal thickness; SRF = subretinal fluid; TCA = total choroidal area. ^a^ *p*-value using paired *t*-test. ^b^ *p*-value using Wilcoxon signed-rank test. Continuous variables are presented as mean ± standard deviation; within-group comparisons between baseline and 6 months were performed using the paired *t*-test. Categorical variables are presented as *n* (%) and were compared using McNemar’s test. A *p*-value < 0.05 was considered statistically significant.

**Table 3:** Comparison of treatment outcomes between the aflibercept 8 mg and faricimab-svoa groups.

	Aflibercept 8 mg (*n* = 32)	Faricimab-svoa (*n* = 34)	*p*-Value
ΔBCVA (LogMAR)	−0.13 ± 0.28	−0.17 ± 0.34	0.534 ^b^
ΔCMT (μm)	−138.2 ± 107.4	−143.4 ± 157.9	0.875 ^a^
ΔSFCT(μm)	−55.2 ± 68.3	−65.8 ± 53.9	0.389 ^a^
ΔHaller layer (μm)	−41.5 ± 43.2	−49.7 ± 38.1	0.452 ^b^
ΔChoriocapillaris/Sattler layer (μm)	−13.8 ± 15.8	−18.9 ± 16.4	0.153 ^b^
ΔCVI	0.018 ± 0.020	0.037 ± 0.027	0.003 **^a^**
ΔTCA (mm^2^)	−0.134 ± 0.182	−0.155 ± 0.156	0.772 ^a^
ΔLCA (mm^2^)	−0.054 ± 0.103	−0.046 ± 0.097	0.818 ^a^
ΔPED height (μm)	−84.5 ± 101.1	−82.5 ± 117.0	0.940 ^b^
Total injection (*n*)	3.53 ± 0.72	3.32 ± 0.68	0.234 ^b^
Dry macula (*n*, %)	24 (75.0%)	27 (79.4%)	0.772

Δ denotes the mean change from baseline to 6 months. BCVA = best-corrected visual acuity; CMT = central macular thickness; CVI = choroidal vascularity index; LCA = luminal choroidal area; LogMAR = logarithm of the minimum angle of resolution; PED = pigment epithelial detachment; TCA = total choroidal area. ^a^ *p*-value using Welch’s *t*-test. ^b^ *p*-value using the Mann–Whitney U test. Continuous variables are presented as mean ± standard deviation and were compared using the independent *t*-test. Categorical variables are presented as *n* (%) and were compared using Fisher’s exact test. A *p*-value < 0.05 was considered statistically significant.

**Table 4:** Comparison of polyp closure rates between aflibercept 8 mg and faricimab-svoa group-subgroup analysis.

	Aflibercept 8 mg (*n* = 17)	Faricimab-svoa (*n* = 17)	*p*-Value
Complete and partial polyp closure	12 (70.6%)	13 (76.5%)	0.702
Complete polyp closure	6 (35.3%)	8 (47.1%)	0.492
Partial polyp closure	6 (35.3%)	5 (29.4%)	0.718
No response	5 (29.4%)	4 (23.5%)	0.702

## Data Availability

The datasets generated during and/or analyzed during the current study are available from the corresponding author on reasonable request.

## Supplementary Materials

The following supporting information can be downloaded at https://www.mdpi.com/article/10.3390/jcm15114355/s1: Figure S1: Measurement of choroidal vascularity index (CVI). (A) Original spectral domain-optical coherence tomography. The total choroidal area (TCA) was manually delineated between the basal margin of the retinal pigment epithelium and the inner scleral boundary (yellow lines). (B) Binarized choroidal image with a modified Niblack local thresholding algorithm. (C) The CVI was calculated as the ratio of luminal choroidal area (LCA) to TCA.

## Abbreviations

The following abbreviations are used in this manuscript:

AMD	Age-related macular degeneration
Ang-2	Angiopoietin-2
BCVA	Best-corrected visual acuity
BUN	Blended RPE-underlying structure
CMT	Central macular thickness
CVI	Choroidal vascularity index
EDI-OCT	Enhanced depth imaging optical coherence tomography
ETDRS	Early Treatment Diabetic Retinopathy Study
ICGA	Indocyanine green angiography
IRF	Intraretinal fluid
LCA	Luminal choroidal area
logMAR	Logarithm of the minimum angle of resolution
MNV	Macular neovascularization
OCT	Optical coherence tomography
OCTA	Optical coherence tomography angiography
PCV	Polypoidal choroidal vasculopathy
PED	Pigment epithelial detachment
PL	Polypoidal lesion
PNV	Pachychoroid neovasculopathy
PRN	Pro re nata
RPE	Retinal pigment epithelium
SFCT	Subfoveal choroidal thickness
SRF	Subretinal fluid
