# Supplementary figures and images for: Comparison of Choroidal Morphological Changes Between Aflibercept 8 mg and Faricimab-svoa in Treatment-Naïve Polypoidal Choroidal Vasculopathy and Pachychoroid Neovasculopathy
Source: J Clin Med. 2026 Jun 4;15(11):4355. doi: 10.3390/jcm15114355 (PMC13257846; doi:10.3390/jcm15114355)

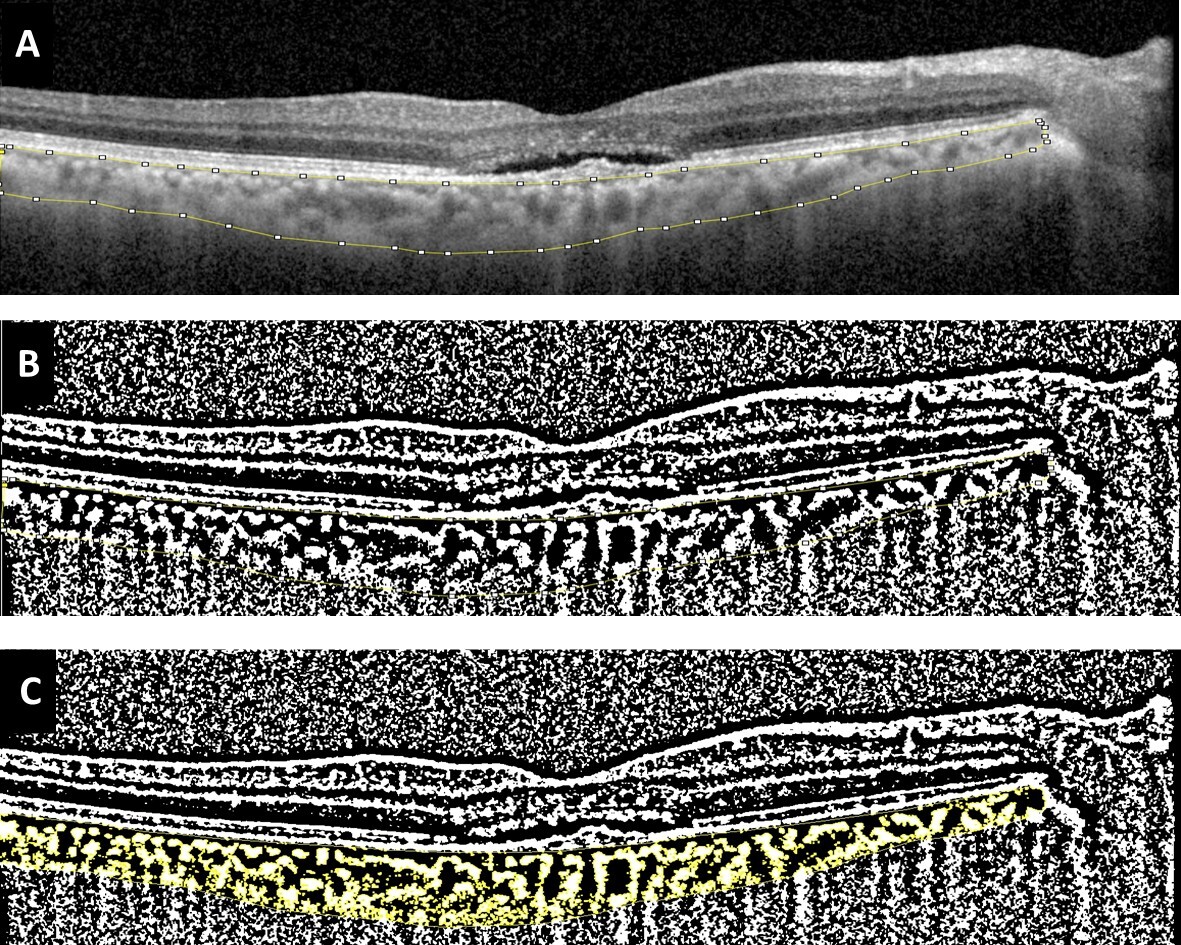

Supplement: Supplementary file 1 [file jcm-15-04355-s001.zip › jcm-4283956-supplementary.jpg]
